# CHALLENGES IN SUPPORTIVE CARE DECISION-MAKING IN THE CONTEXT OF ALZHEIMER’S DISEASE AND RELATED DEMENTIAS

**DOI:** 10.1093/geroni/igae098.3070

**Published:** 2024-12-31

**Authors:** Jean Taylor, Anne Turner

**Affiliations:** University of Washington, Seattle, Washington, United States; University of Washington, Seattle, Washington, United States

## Abstract

In the United States, persons with ADRD face multiple decisions regarding supportive care transitions; however, their involvement in decision-making often declines as their condition progresses. The aim of the Decision Making in Alzheimer’s Research (DMAR) study is to center the voice of persons with dementia by understanding preferences and key factors influencing supportive care decisions. We conducted in-depth online interviews (n=79) with 24 older adults with dementia (aged 65 years and older), 37 informal caregivers, and 18 professionals who work with individuals with dementia. Interviews focused on decision making and identifying factors that related to supportive care decisions. Qualitative analysis of interview transcripts revealed that persons with dementia and caregivers were often not ready to think about or plan for supportive care changes. Challenges participants described in moving forward in planning supportive care included lack of understanding or acceptance of the diagnosis, inability to imagine future scenarios, and lack of openness to planning for the future. Tailored information about the course of dementia and potential future care options are needed. Suggestions by professionals for the best timing for provision of a decision tool ranged from “as soon as possible” to “soon after diagnosis”. Some professionals emphasized the need for ongoing conversations with trusted medical professionals as the disease progresses. Our study highlights the importance of early, tailored, and ongoing support in transition planning. We will share specific suggestions for how and when to implement a decision-making tool to assist with decisions about possible future needs.

